# Pyrosequencing assay for rapid identification of *Mycobacterium tuberculosis *complex species

**DOI:** 10.1186/1756-0500-4-423

**Published:** 2011-10-19

**Authors:** Imen Ben Kahla, Mireille Henry, Jalel Boukadida, Michel Drancourt

**Affiliations:** 1 Laboratoire de Microbiologie et d'Immunologie, UR02/SP13, CHU Farhat Hached Sousse, Tunisie; 2 URMITE, CNRS UMR6236, IRD198, IFR 48, Institut Méditerranée Infection, Aix-Marseille-Université, Marseille, France

**Keywords:** *Mycobacterium tuberculosis *complex, pyrosequencing, identification

## Abstract

**Background:**

Identification of the *Mycobacterium tuberculosis *complex organisms to the species level is important for diagnostic, therapeutic and epidemiologic perspectives. Indeed, isolates are routinely identified as belonging to the *M. tuberculosis *complex without further discrimination in agreement with the high genomic similarity of the *M. tuberculosis *complex members and the resulting complex available identification tools.

**Findings:**

We herein develop a pyrosequencing assay analyzing polymorphisms within *glpK*, *pykA *and *gyrB *genes to identify members of the *M. tuberculosis *complex at the species level. The assay was evaluated with 22 *M. tuberculosis*, 21 *M. bovis*, 3 *M. caprae*, 3 *M. microti*, 2 *M. bovis *BCG, 2 *M. pinnipedii*, 1 *M. canettii *and 1 *M. africanum *type I isolates. The resulted pyrograms were consistent with conventional DNA sequencing data and successfully identified all isolates. Additionally, 127 clinical *M. tuberculosis *complex isolates were analyzed and were unambiguously identified as *M. tuberculosis*.

**Conclusion:**

We proposed a pyrosequencing-based scheme for the rapid identification of *M. tuberculosis *complex isolates at the species level. The assay is robust, specific, rapid and can be easily introduced in the routine activity.

## Background

The *Mycobacterium tuberculosis *complex (MTC) includes *M. tuberculosis*, *M. bovis *and BCG-derived clones, *M. africanum*, *M. canettii*, *M. microti*, *M. caprae *and *M. pinnipedii *[1]. Recently, an eight member named *Mycobacterium mungi *was identified in banded mongooses (*Mungos mungo*) in Botswana [2]. While there is variable host-specificity among the different MTC members, every species, but *M. mungi*, has been implicated in human tuberculosis with *M. tuberculosis *being the most common pathogen [3-6]. The exact contribution of each species in human disease may be underestimated due to the limited capacity of laboratories to identify MTC isolates at the species level in routine practice. Indeed, phenotypic methods are time-consuming and the results are difficult to interpret. Molecular assays are hampered by the high genetic similarity reflected by the complete conservation of the 16S rRNA and *rpoB *genes of the MTC members [7,8]. This situation indeed, led to propose that the various MTC species would be more accurately described as ecotypes [9]. Despite the high degree of nucleotide sequence homology, some genomic markers such as *pncA*, *mpt40*, *hupB*, *gyrB *and *wbbl1*genes in addition to the regions of difference (RD) have been successfully used for the differentiation of MTC members through direct analysis such as in spoligotyping or after enzymatic digestion of PCR products [10-16]. Nevertheless, most of these techniques allow only partial discrimination between the MTC species and are too fastidious to be implemented in the routine practice of microbiology laboratories. The Genotype MTBC (Hain Lifescience, GmbH, Nehren, Germany) is a commercial DNA-strip based assay identifying MTC species through the detection of *gyrB *gene single nucleotide polymorphisms (SNPs) and RD1 [17]. The test is easy to use, but failed to identify *M. canettii *and *M. pinnipeddii *[17].

Pyrosequencing is a real-time non-electrophoretic DNA sequencing technique that can be easily adapted for routine use in the clinical microbiology laboratory. This technique was successfully used for the identification of non-tuberculous mycobacteria, identification of the Beijing family of *M. tuberculosis *and detection of rifampin-resistance among MTC members [18-20]. We here developed a pyrosequencing assay for the rapid identification of the MTC species based on polymorphisms within the *glpK*, *pykA *and *gyrB *genes.

## Methods

### MTC isolates

A set of 8 reference MTC isolates and 47 *M. africanum *type 2 and human and animal clinical isolates representative of all MTC species (excluding *M. mungi ***and *M. africanum *type II) **after molecular identification as previously described [21] was used in this study [15,16,20]. Maria Laura Boschiroli (ANSES, Maisons-Alfort, France) graciously provided DNA extracts of MTC organisms isolated from different animal species (cattle, badger, cat, dog, sea lion, wild boar, wild deer, marmoset and chimpanzee). In addition, 127 clinical MTC organisms identified using ITS-real time PCR were analyzed (Additional file 1). These strains were isolated at the microbiology laboratory of Farhat Hached Hospital, Sousse, Tunisia and the mycobacteria reference laboratory of the Institut Hospitalier Universitaire POLMIT, Marseille, France. This study involves mycobacteria isolated from animals and from patients as a part of the routine diagnostic activity of laboratories and does not involve animals or patients themselves. No informed consent was obtained from individuals in agreement with French law, as the study concerns only microbiota and not the individuals themselves.

### *glpK *and *pykA *gene analyses

The sequence of the *glpK *and *pykA *genes of *M. tuberculosis *H37Rv (Genes ID: 885280, 885501), H37Ra (Genes ID: 5214476, 5215168), F11(Genes ID: 5224418, 5222312), KZN1435 (Genes ID: 8164550, 8160651) and CDC1551(Genes ID: 922690, 924212), *M. bovis *AF2122 (Genes ID: 1093713, 1092578), *M. bovis *BCG Tokyo (Genes ID: 7563752, 7560567) and *M. bovis *BCG Pasteur (Genes ID: 4698362, 4695734) were downloaded from GenBank and aligned using the Clustal software (http://www.clustal.org). In addition, specific primers were designed using the NCBI Primer BLAST (http://www.ncbi.nlm.nih.gov/tools/primer-blast/) for amplification and conventional sequencing of both genes in *M. tuberculosis *(N = 10), *M. bovis *(N = 10), *M. caprae *(N = 3), *M. microti *(N = 3), *M. bovis *BCG (N = 2), *M. pinnipedii *(N = 2), *M. canettii *(N = 1), and *M. africanum *type I (N = 1) isolates (Table 1). PCR reaction was performed in a final volume of 50 μl comprising 22.75 μl of water, 10 μl of 5x Q-buffer (Qiagen, Courtaboeuf, France), 5 μl of 10x buffer, 200 mM of each dDNTP, 1 μl of forward and reverse primers (10 pmol/μl), 2.5 U of hotstar Taq polymearse (Qiagen) and 5 μl of template DNA. Negative controls consisting of reaction mix without DNA were added in each PCR run. The PCR conditions consisted of enzyme activation at 95°C for 15 min, 40 cycles of 30 s at 94°C, 2.5 min at 60°C, 1 min at 72°C and a final elongation at 72°C for 5 min. Sequencing was performed using the BigDye Terminator v1.1 Cycle Sequencing kit (Applied Biosystems, Courtaboeuf, France) and the 3100 genetic analyser (Applied Biosystems) [21]. Sequences were assembled and analysed with the Seqscape software (Applied Biosystems).

**Table 1:** Sequencing and pyrosequencing primers used in this study.

Gene	Method	Primer name	Primer sequence (5' - 3')	Product size (bp)
*glpK*	Sequencing	glpk-Forward	CGACCGCGACTTCCGCAAGT	1,698
		glpk-Reverse	AACATGTCCGCACGCTCGGG	
		glpk-Internal 1	TGATCCGCCGCAAGGCG	
		glpk-Internal 2	CTGGCGGCAAGCTGCAGT	
		glpk-Internal 3	GCTAAACCCGTGTACGCGCT	
		glpk-Internal 4	ATCGCGGTGACCGGCTC	
	Pyrosequencing	glpk-573-F	AGAACGGCGACGCATTGT	106
		glpk-573-R	**Biotin- **CTGGCGTTGGTTACATCGGT	
		glpk-573-Seq	GGTGTTGTGGAATCTGA	
		glpk-845-F	**Biotin- **GGGGAGGCGAAAAACACCTAT	52
		glpk-845-R	CGGTGTTCAGCAGCAGAAAA	
		glpk-845-Seq	AGAAAATTGCCGGTC	
		glpk-1379-F	TCACCGGCAACGACCTGT	126
		glpk-1379-R	**Biotin- **AGAACCCGACCGCCAAGC	
		glpk-1379-Seq	GACCACCGCACTAGG	
*pykA*	Sequencing	pyka-Forward	TACCGCCGTCGCGACTATGC	1,540
		pyka-Reverse	TCGCAAGCGACCTGTTCACCG	
		pyka-Internal 1	CACGATCGGGTGTCCACC	
		pyka-Internal 2	TGGCCGGTGACCGGGTG	
		pyka-Internal 3	TCGATGGCGCCGACGCG	
		pyka-Internal 4	ATGCTGTCCGGGGAAACCT	
	Pyrosequencing	pyka-Forward	GAACTGGTCCACGAGGTGA	100
		pyka-Reverse	**Biotin- **GCACGATCGCTTCGAGATT	
		pyka-Sequencing	CGGTGATCGCCAAGCT	
*gyrB*	Pyrosequencing	gyrB-6307-F	**Biotin- **CGCAAGCTACTGAAGGACAAGG	113
		gyrB-6307-R	TTGGTCTGGCCCTCGAACT	
		gyrB-6307-seq	CCTTCACCGAGATCA	
		gyrB-6406-F	AAGACCAAGTTGGGCAACAC	121
		gyrB-6406-R	**Biotin- **ACACAGCCTTGTTCACAACG	
		gyrB-6406-seq	TGTGCAGAAGGTCTGTAA	

### Pyrosequencing analysis

After analysis of the *glpK *and *pykA *gene sequences, MTC species-specific polymorphic regions were targeted for pyrosequencing [15]. To allow discrimination between all MTC members, single nucleotide polymorphisms (SNPs) within the *gyrB *gene were also used [13]. Specific primers were designed using the PSQ assay (Qiagen) (Table1). In a first step, simplex PCR reactions were performed as described above with 1-min of elongation instead of 2.5 min. PCR products were th**e**n subjected to simplex pyrosequencing analysis using the PyroMark Q96 ID System (Qiagen) as previously described [18]. SNP analyses were performed using the SNP program of the PyroMark Q96 ID software. Sequencing analysis was realized using the sequencing program with a 6(GATC) dispension order. Secondly, we performed multiplex PCR and pyrosequencing reactions with the same conditions as for simplex analysis. The choice of the gene fragments to be simultaneously analyzed was dictated by the PyroMark Q96 ID software so that polymorphic regions did not overlap. For multiplex SNP analysis, two sequencing primers were hybridized either to a single or two PCR amplified loci.

### Specificity testing

A set of clinical isolates of non tuberculous mycobacteria (*Mycobacterium smegmatis*, *Mycobacterium immunogenum*, *Mycobacterium bolletii*, *Mycobacterium chelonae*, *Mycobacterium abscessus*, *Mycobacterium massillense*, *Mycobacterium avium *and *Mycobacterium intracellulare*) and other bacteria (*Escherichia coli*, *Klebsiella pneumoniae*, *Enterococcus feacalis*, *Salmonella enterica, Proteus mirabilis*, *Proteus vulgaris*, *Pseudomonas aeruginosa*, *Citrobacter freundii *and *Stenotrophomonas maltophilia*) were tested on the pyrosequencing analysis to assess the specificity of the assay. Isolates were identified by sequencing the 16S rRNA or the *rpoB *gene and/or using matrix-assisted laser desorption ionization time-of-flight mass spectrometry (Bruker Daltonics, Wissembourg, France) [22].

## Results and discussion

The *pykA *(encoding for the pyruvate kinase) and *glpK *(encoding for the glycerol kinase) genes were previously analyzed to disclose the genetic basis of difference in the glycerol metabolism between *M. tuberculosis *and *M. bovis *in comparison with other MTC species [23,24]. The authors reported a specific gene structure for *M. tuberculosis*, *M. bovis *and *M. bovis *BCG but this particularity has not been further used for identifying MTC organisms. In our study, we extended this previous observation to all MTC species and a larger collection of clinical isolates in the perspective of MTC member's identification.

**Figure 1:**
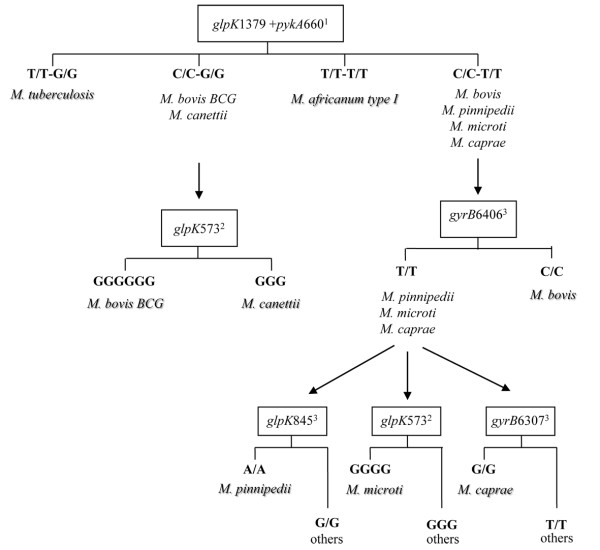
**A proposed scheme for *Mycobacterium tuberculosis *species identification by pyrosequencing**. ^1 ^Genes analysed by multiplex SNP program. ^2^Gene analysed by sequencing program. ^3^Genes analysed by simplex SNP program.

In PCR-based experiments all negative controls remained negative and no PCR products were obtained when testing non-tuberculous mycobacteria and other bacteria confirming the specificity of the primer sets used. After alignment of the *glpK *and *pykA *gene sequences herein achieved and those downloaded from GenBank, we observed MTC species-specific signatures within the *glpK *gene. Four silent SNPs were found at nucleotide (nt) position 279 (A→G), 336 (T→C), 435 (T→A) and 1,497 (T→C) specifically within *M. canettii*. Insertion of one and three guanine at nt 573 was identified in *M. microti *and *M. bovis *BCG, respectively. *M. bovis *reference isolates also shared the same frameshift as *M. microti *at nucleotide 573 in addition to a G→C SNP at nt 779, but these polymorphisms were not found in the human and animal clinical *M. bovis *isolates (N = 10), in agreement with a previous study [23]. Thus, these SNPs further were not considered as *M. bovis *specific feature for subsequent analysis. *M. pinnipedii *specifically exhibits a G→A SNP at nt 845 and *M. tuberculosis *a C→T SNP at nt 1,397. Excluding *M. bovis *reference strains, no specific feature in the *glpK *gene was observed within *M. bovis *and *M. caprae *isolates. As for the *pykA *gene, a unique G→T SNP at nt 660 was commonly detected in *M. bovis*, *M. caprae*, *M. microti *and *M. pinnipedii*. This SNP substitutes glutamic acid with aspartic acid at codon 220, resulting in an inactive pyruvate kinase and the inability of *M. bovis *to use glycerol as a carbone source [23,24]. By analyzing a larger collection of isolates, our results confirmed the presence of this SNP within *M. bovis *and provide the novel evidence for its occurrence also in *M. caprae *and *M. pinnipedii *which were not included in the Keating's study [23]. Accordingly, *M. bovis*, *M. caprae *and *M. pinnipedii *are all characterized by the inability to use glycerol and require pyruvate for growth [24]. We were not able to sequence *glpK *and *pykA *genes in *M. africanum*, nevertheless positive results were obtained by pyrosequencing analysis (see below). Polymorphic regions within the *glpK *and *pykA *genes were targeted for MTC species identification using a pyrosequencing approach. Since no specific signature was obtained for *M. bovis *and *M. caprae*, we combined previously described SNP of the *gyrB *gene to allow complete MTC discrimination [15]. In a first step, the assay was evaluated with the well characterized MTC species (N = 55). The pyrograms obtained from simplex and multiplex data were consistent with the results obtained by conventional DNA sequencing and correctly identifies MTC isolates including 22 *M tuberculosis*, 21 *M. bovis*, 2 *M. bovis *BCG, 3 *M. caprae*, 3 *M. microti*, 2 *M. pinnipeddii*, 1 *M. canettii *and 1 *M. africanum *type I. Accordingly, we propose a generalized diagram for the rapid identification of the MTC species (Figure 1). In a first step, multiplex amplification and pyrosequencing analysis of the *glpK*1379 and *pyk*A660 nt positions in one reaction tube provide four different pyrograms: T/T and G/G specific for *M. tuberculosis*, T/T and T/T specific for *M. africanum *type I, C/C and G/G specific for *M. bovis *BCG and *M. canettii *(group 1) and C/C and T/T specific for *M. bovis*, *M. caprae*, *M. microti *and *M. pinnipedii *(group 2). If pyrogram of group 1 is obtained, sequencing of the *glpK*573 may discriminate between *M. bovis *BCG and *M. canettii*. In the case where pyrogram of group 2 is obtained, analysis of the *gyrB*6406 SNP may specifically identifies *M. bovis *(genotype C/C) from the remaining group 2 species (genotype T/T). *M. pinnipedii *can be identified by SNP analysis of the *glpK*845 (genotype A/A), *M. caprae *by SNP analysis of the *gyrB6307 *and *M. microti *by sequencing the *glpK*573. **These steps can be done in parallel in order to minimize the delay for final identification of any MTC species**. A total of 127 MTC clinical isolates were tested following this scheme and were unambiguously identified as *M. tuberculosis*.

Our proposed scheme allows in one step the specific identification of *M. tuberculosis*, the leading cause of human tuberculosis worldwide and *M. africanum *type I, an important cause of human tuberculosis in west Africa [6,25]. *M. bovis*, an important zoonotic tuberculosis agent and *M. bovis *BCG a safe vaccine exceptionally responsible for disseminated disease in immuno-compromised vaccinated neonates and bladder cancer-patients, could be unambiguously identified in a second reaction after excluding *M. tuberculosis *and *M. africanum *type I [26,27]. The remaining MTC species are less common in human disease and their identification using specific genomic marker described here could be directed by epidemiologic data.

The ability of rapid and accurate identification of MTC members has an important impact both for public health and veterinary facilities. Although a few patients infected with *M. bovis *have been treated by antibiotic regimens incorporating the pyrazinamide, the natural resistance of these species to pyrazinamide makes pyrazinamide generally not recommended for treating such patients [10]. Moreover, it can help epidemiologists and health care professionals to measure the relative contribution of each MTC species in human and animal disease, to identify specific MTC-specie outbreak and to rapidly intercept the impact of the zoonotic transmission particularly in case of *M. bovis *infections so that appropriate control measure could be undertaken.

## Conclusion

Pyrosequencing analysis of the *glpK*, *pykA *and *gyrB *genes provides a robust and easy tool for the rapid identification of all MTC species that can be easily introduced in the routine laboratory activity, **just requiring basics skills in PCR**. The assay allows the simultaneous analyses of up to 96 isolates within 10 to 20 min after **a 4-hour **DNA extraction and PCR amplification **rounds comprising of 3 different PCRs, and 7 different pyrosequencing steps which can be run in parallel**. Moreover, pyrosequencing has several advantages compared to other molecular methods: it determines the exact sequence thereby providing the same accuracy as conventional sequencing methods, the technique dispenses with the need for labeled nucleotides, labeled primers and electrophoresis.

## Competing interests

The authors declare that they have no competing interests.

## Authors' contributions

IBK and MH designed and performed analyses; IBK, JB and MD interpreted data and wrote the draft. All authors read and approved the final manuscript.

## Supplementary Material

Additional file 1 **List of *M. tuberculosis *complex isolates used in this study**. The table lists from left to right the reference number, the identification at the species level, the source, the geographical origin, and the genotype according to Multispacer Sequence Typing of the isolates used in this work. Click here for file
